# Moroctocog Alfa (AF-CC) for Prophylaxis and Treatment of Bleeding Episodes in Previously Treated Patients with Hemophilia A in India

**DOI:** 10.1007/s12288-022-01587-1

**Published:** 2022-11-27

**Authors:** Nirmalkumar Choraria, Savita Rangarajan, M. Joseph John, Shashikant Apte, Pritam Gupta, Seema Pai, Rohit Chand, Shyam Parvatini, G. S. H. Ramakanth, Jeremy Rupon, Amit Chhabra, Hitesh Bhaskarrao Muley, Damien Simoneau

**Affiliations:** 1 Nirmal Hospital Pvt. Ltd, Surat, Gujarat India; 2 K.J. Somaiya Hospital & Research Center, Mumbai, India; 3 University Hospital Southampton NHS Foundation Trust, Southampton, UK; 4 Christian Medical College and Hospital, Ludhiana, India; 5 Pune’s Sahyadri Hospital, Pune, Maharashtra India; 6 Pfizer Healthcare India Pvt. Ltd, Chennai, India; 7 Pfizer Products India Pvt. Ltd, Mumbai, India; 8 Pfizer Inc, Collegeville, PA USA; 9 Pfizer Inc, New York, NY USA; 10 Pfizer IO, Paris, France

**Keywords:** Factor VIII deficiency, Antihemorrhagic, Clinical study, India

## Abstract

**Purpose:**

Hemophilia A is an X-linked congenital disorder, characterized by factor VIII (FVIII) deficiency. Globally, India has the highest population of patients with hemophilia, and there is a clear unmet need for appropriate and effective treatment for this patient population. This multicenter, open-label, post-approval study evaluated the safety and efficacy of moroctocog alfa in patients with moderate or severe congenital hemophilia A in India.

**Methods:**

Intravenous moroctocog alfa was administered 30 ± 5 IU/kg 3 times weekly for bleeding prophylaxis, according to the local product document. Participants were treated for up to 8 weeks, with an up to 4-week screening period and a subsequent post-treatment, 28-day safety observation period. Patients continued in the study until at least 24 exposure days or a period of up to 8 weeks on moroctocog alfa.

**Results:**

A total of 50 participants were enrolled, and 48 (85.7%) completed the study. No participants developed FVIII inhibitors during the study. The mean (SD) annualized bleeding rate during moroctocog alfa prophylaxis was 0.79 (2.0) with a median (range) of 0.00 (0.0, 6.8). The mean (SD) annualized total factor consumption (TFC) per participant was 287,432 (93,866) IU; the mean (SD) annualized TFC by weight per participant was 4176 (858) IU/kg. Moroctocog alfa was well tolerated with no reported treatment-emergent adverse event-related dose reductions, discontinuations, or serious adverse events.

**Conclusion:**

Moroctocog alfa was safe, effective, and well tolerated in Indian participants with congenital moderate to severe hemophilia A. No participant developed FVIII inhibitors during the study.

## Introduction

Hemophilia A is an X-linked congenital disorder, characterized by factor VIII (FVIII) deficiency [1, 2]. Hemophilia A manifests as spontaneous bleeding into the muscles and joints, which, if inadequately managed, becomes painful and destructive to joint architecture [1, 2].

Global survey data from the World Federation of Hemophilia (WFH) indicate that India has the largest population of individuals with hemophilia A worldwide, with 19,690 cases (incidence: 1.4 per 100,000 people) reported in 2020, followed by the United States, with 13,915 cases (4.2 per 100,000 people) [3]. By comparison, the incidence of hemophilia A varies across the Asian region, with 0.8 cases per 100,000 in Indonesia (n = 2214) and 1.1 cases per 100,000 in the Philippines (n = 1155) compared with 2.2 per 100,000 in Thailand (n = 1557) and 3.0 per 100,000 in Malaysia (n = 950) [3].

In India, a large proportion of individuals with hemophilia either remain undiagnosed or present for treatment at a late stage, with many cases unregistered. Consequently, the actual prevalence of hemophilia A is probably underestimated, and when individuals are diagnosed, they typically have more severe disease [4]. Based on these data, India carries a disproportionate amount of the global hemophilia A health care burden, signifying an unmet need for appropriate and effective treatment for this patient population. The current standard of care encompasses prophylactic or on-demand treatment with recombinant or plasma-derived clotting factor concentrate, or prophylaxis with a monoclonal antibody, such as emicizumab [1].

For those with moderate or severe hemophilia A, intravenous replacement with exogenous FVIII is typically used to prevent or treat hemorrhage [1]. Treatment for hemophilia accounts for the majority of the overall cost of management, with clotting factor concentrates accounting for more than 86% of direct costs in individuals without inhibitors [5]. High treatment costs are recognized as a barrier for access to treatment. However, the beneficial quality-of-life outcomes associated with prophylaxis are likely to outweigh the cost of treatment [5–7].

Despite the prevalence of hemophilia A, the use of FVIII (both standard and extended half-life products) is much lower in India than in many other parts of Asia and the rest of the world [3]. Recent 2020 WFH estimates for mean per capita FVIII use in 2019 were 0.289 IU/total population in India compared with estimates ranging from 0.622 IU/total population for Thailand to 2.219 in Singapore, 7.105 in the United States, and 9.026 in the United Kingdom [3]. Moroctocog alfa (AF-CC) is a third-generation recombinant FVIII produced using advanced manufacturing and purification, and is free of exogenous human- and animal-derived protein [8–11]. It is approved in India for the control and prevention of hemorrhagic episodes and for routine and surgical prophylaxis in participants with hemophilia A (congenital FVIII deficiency or classic hemophilia) [12, 13]. This post-approval study evaluated the safety and efficacy of moroctocog alfa in participants with moderate or severe congenital hemophilia A in India.

## Materials and Methods

### Study Design

This single-country, multicenter, open-label, single-arm interventional study was conducted in India between January 25, 2020, and September 24, 2020. Participants were screened for a period of 4 weeks and then treated for up to 8 weeks post-treatment, followed by a 28-day safety observation period. Participants continued in the study until at least 24 exposure days (EDs) or a period of up to 8 weeks on moroctocog alfa (whichever occurred first). Intravenous moroctocog alfa was administered 30 ± 5 IU/kg 3 times weekly for bleeding prophylaxis, according to the local product document (LPD). For on-demand treatment, the amount and frequency of administration of moroctocog alfa were individually tailored according to clinical assessment.

### Participants

Males aged 12 through 65 years with congenital moderate or severe hemophilia A (FVIII activity [FVIII:C] ≤ 5%) and a documented history of at least 50 EDs to FVIII-containing products were included. Participants were excluded if they had a prior history of FVIII inhibitors or positive inhibitor testing (≥ 0.6 Bethesda unit [BU]/mL) during screening, had any bleeding disorder other than hemophilia A, were immunocompromised with HIV, had planned surgery within 6 months of the study initiation, or participated in other studies involving other investigational drugs within 30 days of study initiation.

### End Points

The safety end points were FVIII inhibitor development (≥ 0.6 BU/mL), as confirmed by central laboratory testing (primary end point), and treatment-emergent adverse events (TEAEs), including the incidence of serious adverse events (SAEs), classified according to the Medical Dictionary of Regulatory Activities (MedDRA) v23.1; TEAEs were monitored from the first dose of study drug until treatment end date plus 28 days. Efficacy end points included the annualized bleeding rate (ABR) calculated based on bleeding events that required on-demand infusion with moroctocog alfa while receiving moroctocog alfa as prophylaxis, annualized total factor consumption (TFC) in IUs and by weight (IU/kg), and number of moroctocog alfa infusions required to treat each new bleeding episode.

### Statistical Analysis

Participants who received at least 1 dose of moroctocog alfa were included in the safety analyses. Adverse events (AEs) were summarized using descriptive statistics. Those who received at least 1 dose of moroctocog alfa and had evaluable data for the corresponding end point were included in the efficacy end point analyses; data were summarized using descriptive statistics. The primary endpoint for this study was the proportion of subjects for which FVIII inhibitors were detected at visit 4. Because of the size and nature of this study, a 2-sided 90% confidence interval (CI) was chosen for the corresponding proportion, which was computed using the exact method. For annualized TFC by weight, dose by weight was summed per subject then annualized, i.e. (total units by weight / treatment interval duration) × 365.25, and then used for descriptive statistics calculation. Post hoc analyses computed the proportion of participants who had no treated bleeding events during the prophylaxis period; 95% CIs were calculated, applying the exact method.

Some participants missed moroctocog alfa doses during the study owing to COVID-19 restrictions; bleeding events that may have occurred while participants were not receiving moroctocog alfa prophylaxis were not included in the ABR calculations.

## Results

### Study Population

Of 56 screened participants, 50 were enrolled; of these, 48 (86%) participants completed the study. Two participants (4%) were discontinued during the open-label treatment period: 1 participant withdrew consent during treatment, and 1 completed treatment but did not return for an end-of-treatment evaluation because of COVID-19. All 50 enrolled participants were males of Indian Asian origin, with a mean (standard deviation [SD]) age of 29.6 (9.8) years (Table 1). The mean (SD) number of previous FVIII EDs was 171.5 (240.4) days. A total of 24 (48%) participants had a family history of hemophilia. The majority (49/50, 98%) of participants had no family history of inhibitors to FVIII products. Factor mutation was not classified in any participants, and no participants had a history of inhibitors or allergy to FVIII products.

**Table 1:** Demographics and baseline characteristics (safety analysis population)

Parameter	Results (N = 50)
Age	
Mean (SD), years	29.6 (9.8)
Median (range), years	28 (13 − 61)
Indian race, n (%)	50 (100)
Median (range) body mass index, kg/m^2^	24.4 (14.9 − 32.8)
Factor VIII activity (%), median (range)	0.99 (1–12)^a^
Mean (SD) laboratory values	
ALT (U/L)	0.596 (0.4411)
AST (U/L)	0.439 (0.2252)
Bilirubin (µmol/L)	11.75 (6.257)
ALP (U/L)	1.853 (0.7785)

ALP, alkaline phosphatase; ALT, alanine aminotransferase; AST, aspartate aminotransferase; FVIII:C, factor VIII activity; SD, standard deviation

^a^Two participants did not meet the inclusion criterion of FVIII:C ≤ 5%; their baseline values were 5.3% and 12%. Prior lab reports noted that they had FVIII:C ≤ 5%; however, they were supplemented with FVIII just before screening, which increased their FVIII:C activity at screening

The total mean (SD) treatment interval duration was 68.2 (26.3) days (median [range]: 59.5 [45.0, 183.0]). The total mean (SD) EDs per participant during the study was 23.9 (0.6) days (median [range]: 24.0 [20.0, 24.0]), and the mean (SD) EDs per participant for prophylaxis was 23.8 (0.6) days (median [range]: 24.0 [20.0, 24.0]). FVIII other than moroctocog alfa was received by 22 (44%) participants for the treatment of bleeding episodes that occurred during follow-up. Because of COVID-19 restrictions, 1 participant received nonstudy treatment during the treatment period for a bleeding event, per investigator discretion.

### Safety

Three (6%) participants experienced 6 TEAEs (Table 2). All cases were mild in severity, and none were considered by the investigators to be related to moroctocog alfa. One participant reported an AE of arthralgia during the follow-up period, which was not treatment-emergent. No TEAE-related dose reductions, discontinuations, and SAEs were reported.

**Table 2:** Treatment-emergent adverse events

Parameter	Results (N = 50)
Number of participants with AEs, n (%)	3 (6)
TEAEs, n (%)	3 (6)
SAEs, n (%)	0 (0)
Withdrawals because of AEs, n (%)	0 (0)
AEs^a^, n (%)	
Arthralgia	1 (2)
Diarrhea	1 (2)
Hypertension	1 (2)
Pyrexia	1 (2)
Vertigo	1 (2)
Vomiting	1 (2)

AE, adverse event; SAE, serious adverse event; TEAE, treatment-emergent adverse event

^a^If the same participant in a given treatment had more than 1 occurrence in the same preferred term event category, only the most severe occurrence was counted. Participants are counted only once per treatment per event

### Clinical Outcomes

No participants developed FVIII inhibitors (≥ 0.6 BU/mL) during the course of the study, as confirmed by central laboratory testing. The upper and lower bounds of the 2-sided 90% CI for the proportion of participants for which FVIII inhibitors were detected at visit 4 were 0.00% and 0.06%, respectively, confirming that the likelihood of a participant developing FVIII inhibitors is < 0.1%. A total of 7 (14%) participants reported a single bleeding event (5 [10%] spontaneous and 2 [4%] traumatic) each requiring a single on-demand moroctocog alfa infusion (Table 3). The mean (SD) ABR during moroctocog alfa prophylaxis was 0.79 (2.0) with a median (range) of 0.00 (0.0, 6.8).

**Table 3:** Details of bleeding episodes and hemostatic response to moroctocog alfa

Parameter	Results (N = 50)
Number of participants with bleeding events, n (%)^a^	7 (14)
ABR^b,c^	
Mean (SD)	0.79 (2.0)
Median (range)	0.0 (0.0 − 6.8)
ABR according to bleeding site, n (%)^a^	
Joint	7 (14)
Soft tissue/muscle	0 (0)
Multiple	0 (0)
Other	0 (0)
ABR according to bleeding type, n (%)^a^	
Spontaneous	5 (10)
Traumatic	2 (4)
Procedural	0 (0)
Number of infusions to treat each new bleeding event	
Mean (SD)	1.0 (0.0)
Median (range)	1.0 (1.0 − 1.0)
Annualized TFC^d^	
Per participant, mean (SD), IU	287,432.3 (93,866.2)
By weight, mean (SD), IU/kg	4175.7 (858.4)

ABR, annualized bleeding rate; SD, standard deviation; TFC, total factor consumption

^a^Number of subjects with bleeding events requiring and receiving on-demand treatment with moroctocog alfa during the treatment interval duration

^b^ABR = (number of bleeding events / treatment interval duration) × 365.25

^c^Treatment interval duration = (date of visit 4 – date of visit 2 + 1); if the date of visit 4 – last dosing date was > 10, then the date of visit 4 was replaced with the last dosing date; if the date of visit 4 was missing (due to discontinuation or any other reason), then the date of visit 4 was replaced with the last dosing date or the last participant visit date, whichever was greater

^d^Annualized TFC = (TFC / treatment interval duration) × 365.25

A post hoc analysis showed that during prophylaxis, 42/50 (84%) participants had no bleeding episodes that required treatment. Seven participants each had 1 bleeding episode that was treated with moroctocog alfa. One participant had a bleeding event and received nonstudy treatment during the treatment interval duration owing to COVID-19 restrictions.

All but 1 of the 77 bleeding episodes that occurred during the follow-up period (after the end-of-treatment visit) were spontaneous, and most were treated with other FVIII treatment given on the same day, or 1 day after the bleeding event. During the treatment period, 7 participants had a bleeding event (1 each), but none of them discontinued prophylaxis. The mean (SD) annualized TFC per participant was 287,432 (93,866) IU; the mean (SD) annualized TFC by weight per participant was 4176 (858) IU/kg (Table 3).

## Discussion

This phase 4 study confirms the safety and efficacy of moroctocog alfa in Indian participants with moderate to severe hemophilia A receiving prophylaxis with moroctocog alfa for 8 weeks, which corresponded to a minimum of 24 EDs. Moroctocog alfa was generally well tolerated, with only 4 (8%) participants reporting an AE, of which 3 (6%) were treatment-emergent AEs. All AEs were mild, and none were considered related to treatment. No participant developed FVIII inhibitors (≥ 0.6 BU/mL), as confirmed by central laboratory testing.

Among participants receiving moroctocog alfa for a duration of 8 weeks, the mean ABR was < 1 (0.79). All 7 bleeding episodes observed during the study and included in the analysis were effectively managed with a single on-demand infusion of moroctocog alfa. Of note, 24 participants were receiving on-demand treatment prior to enrollment and initiation of short-term prophylaxis in this study; despite this, no participant developed inhibitors, and few experienced bleeding events while receiving prophylaxis with moroctocog alfa.

There is a clear unmet need for effective prophylactic regimens in India, which has the largest number of participants with hemophilia A globally but with relatively low per capita use of FVIII treatment [3]. In the current study, moroctocog alfa was effective for the control and prevention of hemorrhagic episodes, with a mean ABR of less than 1.0 observed, and a single on-demand infusion successfully treated participants who experienced a bleeding event.

Further analyses to determine whether a reduction in the number of bleeding events, along with the consequent reduction in the need for on-demand infusions, could potentially diminish overall health care burden and enhance patients’ quality of life would be beneficial. In addition, further study is required to determine whether transition from an on-demand regimen to a prophylaxis regimen could allay concerns regarding bleeding risk, and potentially lead to an increase in FVIII activity levels and a decrease in bleeding events. The relatively short 8-week duration of the current study did not allow for long-term collection of meaningful data on the efficacy of bleeding event prevention and quality-of-life outcomes. Nevertheless these findings provide valuable insight on the efficacy of moroctocog alfa in an Indian population with hemophilia A.

In conclusion, moroctocog alfa was safe and effective in Indian participants with congenital moderate to severe hemophilia A. No participant developed FVIII inhibitors during the study, and the safety data were consistent with the known safety profile of moroctocog alfa. Moroctocog alfa was effective for controlling and preventing hemorrhagic episodes when used according to the LPD.

## Data Availability

Upon request, and subject to review, Pfizer will provide the data that support the findings of this study. Subject to certain criteria, conditions, and exceptions, Pfizer may also provide access to the related individual de-identified participant data. See https://www.pfizer.com/science/clinical-trials/trial-data-and-results for more information.
